# Sarcopenia independently predicts acute cholecystitis in older patients with gallstones: a retrospective cohort study

**DOI:** 10.3389/fmed.2026.1804441

**Published:** 2026-04-13

**Authors:** Hua Fan, Di Wang, Huaqing Liu, Zhenghao Pei, Jun Wang, Jialin Zhang, Shangxin Wang, Gengze Wang

**Affiliations:** 1 Department of Gastroenterology & General Surgery, Nanyang Central Hospital, Nanyang, Henan, China; 2 Department of Minimally Invasive Surgery, Nanyang Central Hospital, Nanyang, Henan, China

**Keywords:** acute cholecystitis (AC), gallstones, older adults, risk factor (RF), sarcopenia

## Abstract

**Background:**

This study investigated whether sarcopenia independently predicts incident acute cholecystitis (AC) in geriatric patients with gallstones.

**Methods:**

This retrospective cohort study included 1,177 patients aged ≥65 years with incidentally detected gallstones and no prior biliary disease. Sarcopenia was diagnosed using Asian Working Group for Sarcopenia 2019 criteria. The primary outcome was AC development during follow-up, adjudicated using Tokyo Guidelines 2018. Multivariate logistic and Cox regression models adjusted for age, BMI, sex, alcoholism, and Charlson Comorbidity Index.7

**Results:**

During follow-up, 336 patients (28.5%) developed AC. The AC group exhibited significantly higher sarcopenia prevalence (38.10 vs. 14.03%; *p* < 0.001) and lower mean handgrip strength (22.92 ± 12.51 vs. 25.50 ± 12.44 kg; *p* < 0.001) and appendicular skeletal muscle mass (6.82 ± 2.74 vs. 7.67 ± 2.93 kg/m^2^; *p* < 0.001) compared to controls. Sarcopenia independently increased AC risk by 3.56-fold (*OR* = 3.564, 95%CI: 2.619–4.860; *p* < 0.001) and AC hazard by 2.42-fold (*HR* = 2.419, 95%CI: 1.912–3.060; *p* < 0.001). Kaplan-Meier analysis demonstrated significantly reduced AC-free survival in sarcopenic patients (log-rank *p* < 0.001). Severe sarcopenia did not confer additional risk beyond standard sarcopenia criteria.

**Conclusions:**

Sarcopenia is a robust, independent predictor of AC development in older adults with gallstones, offering superior risk stratification compared to adiposity-based metrics. Routine sarcopenia assessment may identify high-risk individuals for targeted preventive interventions.

## Introduction

Cholelithiasis represents a major public health burden, affecting 10%−20% of the adult population globally (1). While the majority of patients remain asymptomatic, approximately 1%−3% of those with gallstones develop acute cholecystitis (AC) annually, accounting for 3%−10% of all emergency admissions for acute abdominal pain (2, 3). The progression from silent gallstones to AC is unpredictable, and current clinical practice lacks reliable tools to stratify high-risk individuals for proactive management (1, 4).

Traditional risk factors for gallstone disease—the “4Fs” (Female, Fat, Forty, and Fertile) and Body Mass Index (BMI)—have well-established limitations in predicting disease progression (5, 6). BMI, in particular, is a crude anthropometric measure that fails to discriminate between metabolically distinct compartments, such as visceral adiposity and skeletal muscle mass, and does not reflect functional status (7–9). This underscores the urgent need for more precise and physiologically relevant predictors that capture the complex interplay between metabolic health and inflammatory susceptibility.

Sarcopenia, defined as the age-related loss of skeletal muscle mass, strength, and physical performance, has emerged as a robust prognostic factor across diverse medical and surgical conditions (10). The Asian Working Group for Sarcopenia 2019 (AWGS 2019) criteria provide a standardized diagnostic framework that integrates three core domains (11): muscle strength, physical performance, and appendicular skeletal muscle mass (ASM). This multidimensional approach captures not only muscle quantity but also functional reserve. The pathophysiological relevance of sarcopenia extends beyond frailty; it is intrinsically linked to chronic low-grade systemic inflammation, reduced immunocompetence, and impaired stress response capacity (12). These mechanisms may lower the inflammatory threshold required for an obstructing gallstone to trigger acute gallbladder wall inflammation.

While prior research has demonstrated that sarcopenia predicts adverse outcomes after AC develops, including increased length of stay, ICU admission, and mortality, its role in the initial transition from asymptomatic cholelithiasis to AC remains unexplored (13). Sarcopenia is characterized by chronic low-grade systemic inflammation, with elevated circulating levels of pro-inflammatory cytokines including interleukin-6 (IL-6) and tumor necrosis factor-α (TNF-α), and this pro-inflammatory milieu may prime the gallbladder mucosa, lowering the threshold for an exaggerated inflammatory cascade when mechanical obstruction by a gallstone occurs (14–16). We hypothesize that patients with sarcopenia possess diminished physiological reserve and a pro-inflammatory milieu that predisposes to AC when challenged by mechanical obstruction. Furthermore, the comprehensive assessment mandated by AWGS 2019 may identify a high-risk phenotype that is missed by imaging-based body composition analysis alone.

This large-scale retrospective cohort study, comprising 1,177 patients aged ≥65 years with incidentally detected, previously uncomplicated gallstones and a minimum 12-months follow-up, aimed to determine whether sarcopenia, assessed using the rigorous AWGS 2019 diagnostic algorithm, independently predicts the development of AC. By investigating this relationship in a geriatric population uniquely vulnerable to both sarcopenia and biliary complications, we seek to establish a novel risk stratification paradigm that shifts the focus from simple adiposity to holistic muscle health and functional capacity, potentially enabling targeted preventive interventions in at-risk older adults.

## Methods

### Study design and ethics statement

This single-center retrospective cohort study was conducted at Nanyang Central Hospital and approved by the Institutional Review Board (No. 20200079). Written informed consent was obtained from all patients for the use of their clinical data in research. The study protocol adhered to the principles of the Declaration of Helsinki and complied with institutional ethical guidelines.

### Patient population

We retrospectively identified patients aged ≥65 years who underwent abdominal computed tomography (CT) or ultrasonography between May 2020 and May 2025 and were incidentally found to have gallstones. Patients were included if they: (1) were ≥65 years of age; (2) had incidentally detected gallstones documented on baseline imaging; (3) had no history of acute cholecystitis, biliary colic, or cholecystectomy prior to the index imaging; (4) had documented assessments of muscle strength, physical performance, and muscle mass; and (5) either developed acute cholecystitis during follow-up (AC group) or, for those who did not, had a minimum follow-up period of 12 months from the index imaging (control group). Exclusion criteria were: (1) acalculous cholecystitis; (2) malignant biliary obstruction; (3) incomplete baseline sarcopenia assessment data; and (4) loss to follow-up before meeting the criteria for their respective group.

### Sarcopenia assessment

Sarcopenia was diagnosed and categorized according to the Asian Working Group for Sarcopenia 2019 (AWGS 2019) criteria, which require the presence of low muscle mass plus low muscle strength or low physical performance (11).

Muscle Strength: handgrip strength (HGS) was measured using a calibrated spring-loaded dynamometer with the patient seated, elbow flexed at 90°, and forearm in a neutral position. The maximum value from three consecutive measurements of the dominant hand was recorded. Low muscle strength was defined as HGS < 28 kg for men and < 18 kg for women.

Physical Performance: the Short Physical Performance Battery (SPPB) was administered by trained geriatric nurses, consisting of three components: balance tests, 4-meter gait speed, and five repeated chair stands. Low physical performance was defined as an SPPB total score ≤ 9 points.

Muscle Mass: appendicular skeletal muscle mass (ASM) was measured via bioelectrical impedance analysis (InBody BWA2.0, Seoul, Korea), depending on clinical availability. ASM was normalized to height to account for adiposity. Low muscle mass was defined as ASM index < 7.0 kg/m^2^ in men, < 5.7 kg/m^2^ in women, in accordance with AWGS 2019 cutoffs for Asian populations.

Patients meeting criteria for low muscle mass and low muscle strength (or low physical performance) were diagnosed with sarcopenia. Those exhibiting low muscle strength, low muscle mass, and low physical performance simultaneously were classified as having severe sarcopenia.

### Outcome definition

The primary outcome was the development of acute cholecystitis (AC) during follow-up. AC was diagnosed based on the Tokyo Guidelines 2018 (TG18) criteria, requiring fulfillment of at least one item from each of the following three categories: (1) local signs of inflammation (Murphy's sign or right upper quadrant pain/tenderness), (2) systemic signs of inflammation (fever, elevated C-reactive protein, or leukocytosis), and (3) characteristic imaging findings (gallbladder wall thickening >3 mm, pericholecystic fluid, or sonographic Murphy's sign). All cases were independently adjudicated by two gastroenterologists blinded to sarcopenia status; discrepancies were resolved by a third senior reviewer.

### Data collection

Patients were followed retrospectively from the date of index imaging until the development of AC, cholecystectomy, death, or the end of the study period. To ensure adequate observation, a minimum follow-up of 12 months was required for individuals in the control group. Baseline clinical, anthropometric, and laboratory data were systematically extracted from electronic medical records, including: age (years), BMI (kg/m^2^), sex (female or male), smoking history (yes or no), alcoholism history (yes or no), Charlson Comorbidity Index (CCI) score >4 (yes or no), electrocardiogram abnormalities (yes or no), chest radiograph abnormalities (yes or no), hypertension (yes or no), red blood cell count (RBC, 10^12^/L), hemoglobin (Hb, g/L), serum albumin (ALB, g/L), fasting glucose (GLU, mmol/L), and C-reactive protein (CRP, mg/L).

### Statistical analysis

Categorical variables were presented as frequencies and percentages, and continuous variables as mean ± standard deviation (SD). Distribution normality was assessed by the Shapiro–Wilk test. Baseline characteristics were compared between sarcopenic and non-sarcopenic groups using the chi-square test for categorical variables and independent t-test or Mann–Whitney U-test for continuous variables. Time-to-event analysis was performed with the primary endpoint being time to AC development. Cumulative AC-free survival probabilities were estimated using the Kaplan-Meier method, and survival curves were compared between sarcopenic and non-sarcopenic groups using the log-rank test. Univariate logistic regression was performed to identify potential predictors of AC. Variables with p < 0.10 in baseline characteristics comparison were included in multivariate model 1 (age, BMI, and alcoholism history), and these variables along with clinically relevant covariates (sex and CCI) were entered into multivariate logistic regression model 2 to determine whether sarcopenia independently predicted AC development. Odds ratios (OR) with 95% confidence intervals (CI) were reported. Univariate and multivariate Cox proportional hazards regression models were constructed to using the same strategy. Hazard ratios (HR) with 95% confidence intervals (CI) were reported. In addition to binary variable (non-sarcopenia and sarcopenia), sarcopenia status was analyzed as a three-category variable (non-sarcopenia, sarcopenia, severe sarcopenia) with sarcopenia as the reference category to test whether severe sarcopenia conferred incremental risk beyond standard diagnostic thresholds. Statistical significance was set at p < 0.05. All analyses were performed using R software (v4.2.0) for survival analysis.

## Results

### Patient population and follow-up

The study cohort comprised a total of 1,177 patients aged ≥65 years with incidentally detected gallstones, who were divided into two groups: the AC group (*n* = 336), who developed acute cholecystitis during follow-up, and the Normal group (*n* = 841), who did not. To ensure adequate observation for the control group, a minimum follow-up of 12 months was required. The overall sample had a mean age of 75.97 ± 6.86 years and was predominantly female (54.97%).

The demographic, clinical, and laboratory characteristics of both groups are summarized in Table 1. The mean age of the AC group was significantly higher than that of the Normal group (76.83 ± 6.72 years vs. 75.62 ± 6.88 years; *p* = 0.004). Patients in the AC group also demonstrated a higher mean BMI compared to the Normal group (24.58 ± 6.19 kg/m^2^ vs. 22.93 ± 5.60 kg/m^2^; *p* < 0.001). The prevalence of alcoholism history was significantly greater in the AC group (18.15 vs. 13.08%; *p* = 0.032).

**Table 1:** Baseline characteristics of populations included in our study.

Variables	Overall	Normal	AC	*p*-value
	**(*****n*** = **1,177)**	**(*****n*** = **841)**	**(*****n*** = **336)**	
Age (years)	75.97 ± 6.86	75.62 ± 6.88	76.83 ± 6.72	0.004
BMI (kg/m^2^)	23.40 ± 5.82	22.93 ± 5.60	24.58 ± 6.19	< 0.001
Sex (female)	647 (54.97%)	468 (55.65%)	179 (53.27%)	0.5
Smoking history (yes)	270 (22.94%)	196 (23.31%)	74 (22.02%)	0.692
Alcoholism history(yes)	171 (14.53%)	110 (13.08%)	61 (18.15%)	0.032
CCI score (>4)	362 (30.76%)	268 (31.87%)	94 (27.98%)	0.216
Electrocardiogram (abnormal)	315 (26.76%)	222 (26.40%)	93 (27.68%)	0.707
Chest radiograph (abnormal)	252 (21.41%)	178 (21.17%)	74 (22.02%)	0.806
Hypertension (yes)	416 (35.34%)	286 (34.01%)	130 (38.69%)	0.147
RBC (10^12^/L)	4.37 ± 1.43	4.35 ± 1.43	4.41 ± 1.44	0.718
Hb (g/L)	87.80 ± 26.26	87.39 ± 26.27	88.83 ± 26.25	0.418
ALB (g/L)	36.09 ± 13.29	35.87 ± 13.23	36.66 ± 13.44	0.193
GLU (mmol/L)	5.32 ± 1.95	5.29 ± 1.91	5.39 ± 2.04	0.714
CRP (mg/L)	11.90 ± 43.40	12.19 ± 45.95	11.18 ± 36.29	0.788

AC, acute cholecystitis; BMI, body mass index; CCI, Charlson Comorbidity Index; RBC, red blood cell count; Hb, hemoglobin; ALB, serum albumin; GLU, fasting glucose; CRP, C-reactive protein.

No statistically significant differences emerged between the groups regarding sex distribution (53.27 vs. 55.65% female; *p* = 0.500), smoking history (22.02 vs. 23.31%; *p* = 0.692), CCI score >4 (27.98 vs. 31.87%; *p* = 0.216), electrocardiogram abnormalities (27.68 vs. 26.40%; *p* = 0.707), chest radiograph abnormalities (22.02 vs. 21.17%; *p* = 0.806), or hypertension (38.69 vs. 34.01%; *p* = 0.147).

Similarly, baseline laboratory parameters were comparable between the two groups, including red blood cell count (4.41 ± 1.44 vs. 4.35 ± 1.43 × 10^12^/L; *p* = 0.718), hemoglobin (88.83 ± 26.25 vs. 87.39 ± 26.27 g/L; *p* = 0.418), serum albumin (36.66 ± 13.44 vs. 35.87 ± 13.23 g/L; *p* = 0.193), fasting glucose (5.39 ± 2.04 vs. 5.29 ± 1.91 mmol/L; *p* = 0.714), and C-reactive protein (11.18 ± 36.29 vs. 12.19 ± 45.95 mg/L; *p* = 0.788).

### Sarcopenia metrics and comparison between groups

Comprehensive assessment of sarcopenia components revealed significant disparities between the two cohorts (Table 2). The AC group exhibited markedly reduced muscle strength, with mean handgrip strength (HGS) significantly lower than the Normal group (22.92 ± 12.51 kg vs. 25.50 ± 12.44 kg; *p* < 0.001). Similarly, appendicular skeletal muscle mass (ASM) was substantially decreased in the AC group (6.82 ± 2.74 kg/m^2^ vs. 7.67 ± 2.93 kg/m^2^; *p* < 0.001). Physical performance, quantified by the Short Physical Performance Battery (SPPB), was also diminished in the AC group, though the absolute difference was modest (9.18 ± 2.75 vs. 9.59 ± 2.56; *p* = 0.01).

**Table 2:** Sarcopenia metrics between normal and AC groups.

Variables	Overall	Normal	AC	*p*-value
	**(*****n*** = **1,177)**	**(*****n*** = **841)**	**(*****n*** = **336)**	
HGS (kg)	24.77 ± 12.51	25.50 ± 12.44	22.92 ± 12.51	< 0.001
ASM (kg/m^2^)	7.43 ± 2.90	7.67 ± 2.93	6.82 ± 2.74	< 0.001
SPPB (scores)	9.47 ± 2.62	9.59 ± 2.56	9.18 ± 2.75	0.01
Low HGS (yes)	539 (45.79%)	349 (41.50%)	190 (56.55%)	< 0.001
Low ASM (yes)	447 (37.98%)	286 (34.01%)	161 (47.92%)	< 0.001
Low SPPB (yes)	357 (30.33%)	227 (26.99%)	130 (38.69%)	< 0.001
Sarcopenia (yes)	246 (20.90%)	118 (14.03%)	128 (38.10%)	< 0.001
Sarcopenia status				< 0.001
Non-sarcopenia	931 (79.10%)	723 (85.97%)	208 (61.90%)	
Sarcopenia	121 (10.28%)	57 (6.78%)	64 (19.05%)	
Severe sarcopenia	125 (10.62%)	61 (7.25%)	64 (19.05%)	

HGS, handgrip strength; ASM, Appendicular Skeletal Muscle Mass Index; SPPB, Short Physical Performance Battery.

When applying the AWGS 2019 diagnostic thresholds, the prevalence of all sarcopenia components was significantly elevated in the AC group. Low muscle strength affected 56.55% of AC patients compared to 41.50% of controls (*p* < 0.001). Low muscle mass was present in 47.92% of the AC group vs. 34.01% of the Normal group (*p* < 0.001). Low physical performance was identified in 38.69% of AC patients compared to 26.99% of controls (*p* < 0.001). Overall, sarcopenia prevalence was nearly three-fold higher in the AC group (38.10 vs. 14.03%; *p* < 0.001). The distribution of sarcopenia severity also differed markedly (*p* < 0.001), with both sarcopenia (19.05 vs. 6.78%) and severe sarcopenia (19.05 vs. 7.25%) being substantially more prevalent among those who developed AC.

**Figure 1:**
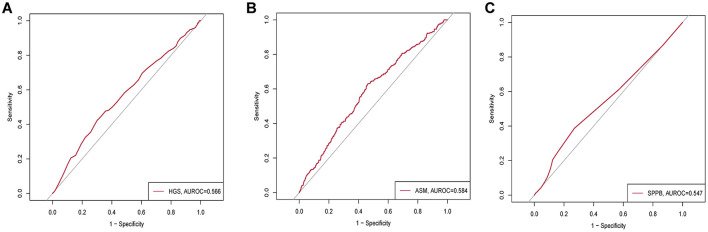
Receiver operating characteristic (ROC) curves for individual sarcopenia components in predicting acute cholecystitis. **(A):** HGS; **(B):** ASM; **(C):** SPPB.

We evaluated the individual predictive performance of each sarcopenia component using ROC curve analysis (Figure 1). Handgrip strength demonstrated modest discriminative ability (AUROC = 0.566). Appendicular skeletal muscle mass exhibited similar performance (AUROC = 0.584). The SPPB score showed slightly lower discriminative capacity (AUROC = 0.547). These findings indicate that while each individual sarcopenia metric was significantly associated with AC development in univariate analysis, their standalone predictive utility for identifying high-risk patients was limited.

### Kaplan-Meier survival analysis

**Figure 2:**
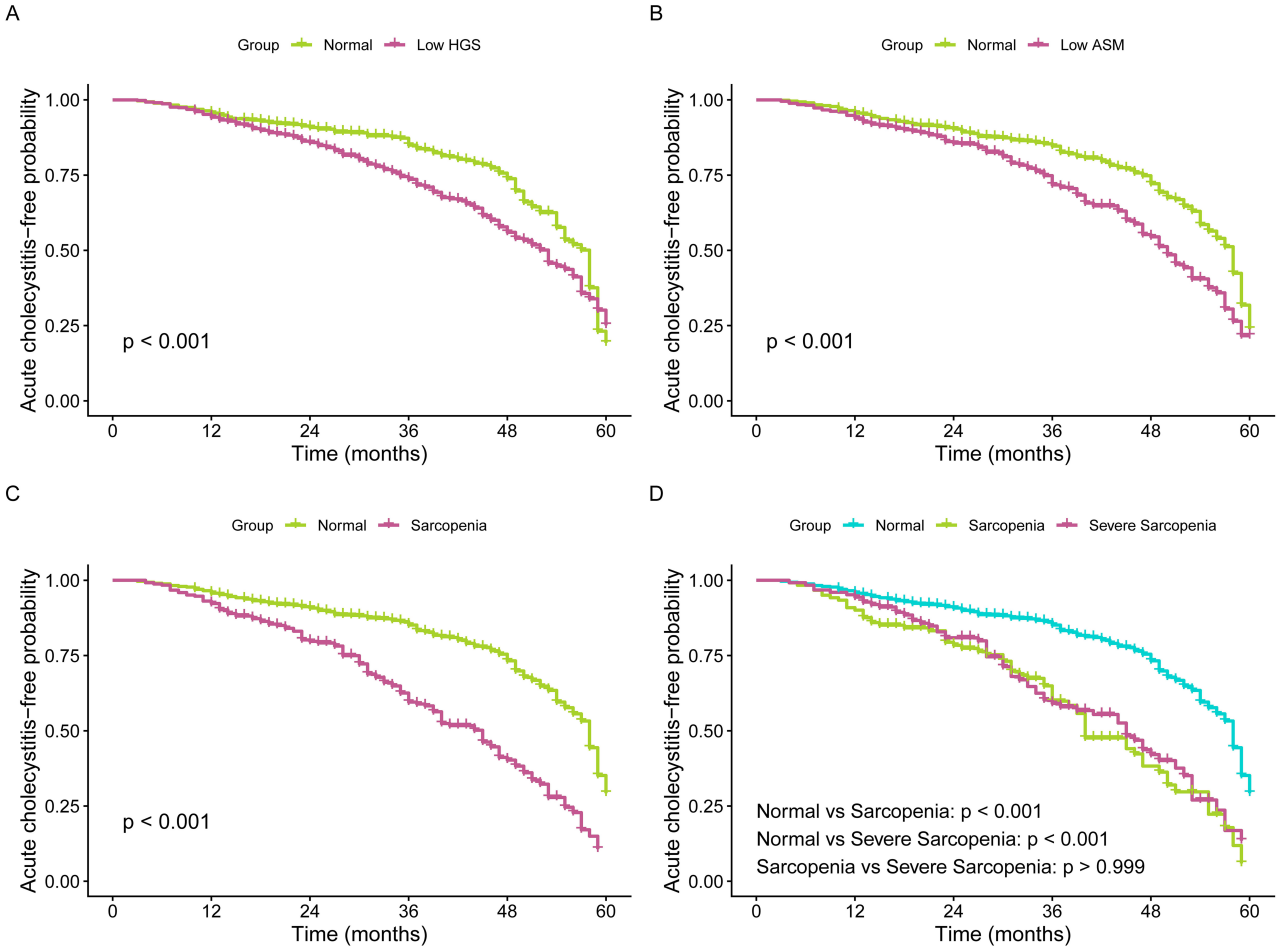
Kaplan-Meier curves of acute cholecystitis-free survival stratified by sarcopenia status. **(A):** low vs. normal handgrip strength; **(B):** low vs. normal appendicular skeletal muscle mass; **(C):** sarcopenia vs. non-sarcopenia status; **(D):** severe sarcopenia vs. sarcopenia vs. non-sarcopenia.

We performed Kaplan-Meier analysis to compare AC-free survival probabilities according to sarcopenia status and its individual components (Figure 2). Patients with low muscle strength exhibited significantly reduced AC-free survival compared to those with normal HGS (log-rank *p* < 0.001) (Figure 2A). Similarly, individuals with low ASM demonstrated markedly inferior survival outcomes (log-rank *p* < 0.001) (Figure 2B). When stratifying by overall sarcopenia status, we observed profound differences in AC-free survival (log-rank *p* < 0.001) (Figure 2C). Both sarcopenia and severe sarcopenia were associated with significantly worse survival compared to non-sarcopenic individuals (both pairwise *p* < 0.001). Notably, there was no significant difference in AC-free survival between patients with sarcopenia and those with severe sarcopenia (pairwise *p* > 0.999), indicating that the presence of either condition conferred similarly elevated risk.

### Logistic regression analysis

To identify independent predictors of AC development, we constructed multivariate logistic regression models adjusting for potential confounders. In univariate analysis, all sarcopenia components demonstrated significant associations with AC (Table 3). The results of co-variables in univariate analysis were summarized in Supplementary Table 1. Handgrip strength, ASM, and SPPB scores as continuous variables exhibited protective effects, with each unit increase associated with a 1.7, 10.0, and 5.6% reduction in AC risk, respectively (all *p* < 0.05). Dichotomous measures of low muscle function showed even stronger relationships, with low HGS, low ASM, and low SPPB each conferring approximately 1.7–1.8-fold increased odds of AC (all *p* < 0.001).

**Table 3:** Logistic regression analysis of sarcopenia metrics for acute cholecystitis.

Variables	Univariate model		Multivariate model 1		Multivariate model 2	
	**OR [95 %CI]**	***P***	**OR [95 %CI]**	***p***	**OR [95 %CI]**	***p***
HGS (continuous)	0.983 [0.972, 0.993]	0.001	0.986 [0.975, 0.996]	0.008	0.985 [0.974, 0.996]	0.006
ASM (continuous)	0.900 [0.859, 0.942]	< 0.001	0.907 [0.866, 0.950]	< 0.001	0.908 [0.866, 0.951]	< 0.001
SPPB (continuous)	0.944 [0.902, 0.989]	0.014	0.950 [0.906, 0.996]	0.032	0.948 [0.904, 0.994]	0.025
Low HGS (yes)	1.835 [1.422, 2.371]	< 0.001	1.747 [1.349, 2.267]	< 0.001	1.766 [1.353, 2.310]	< 0.001
Low ASM (yes)	1.785 [1.380, 2.310]	< 0.001	1.678 [1.291, 2.180]	< 0.001	1.682 [1.289, 2.197]	< 0.001
Low SPPB (yes)	1.707 [1.306, 2.229]	< 0.001	1.609 [1.225, 2.111]	0.001	1.626 [1.237, 2.135]	< 0.001
Sarcopenia (yes)	3.771 [2.812, 5.064]	< 0.001	3.467 [2.564, 4.696]	< 0.001	3.564 [2.619, 4.860]	< 0.001
Sarcopenia status						
Non-sarcopenia	0.256 [0.173, 0.378]	< 0.001	0.267 [0.179, 0.396]	< 0.001	0.264 [0.176, 0.394]	< 0.001
Sarcopenia	Ref	Ref	Ref	Ref	Ref	Ref
Severe sarcopenia	0.934 [0.566, 1.542]	0.791	0.855 [0.513, 1.423]	0.547	0.883 [0.528, 1.472]	0.633

Model 1 adjusted for age, BMI, and alcoholism history. Model 2 additionally adjusted for sex and Charlson Comorbidity Index score. OR, odds ratio; CI, confidence interval; HGS, handgrip strength; ASM, Appendicular Skeletal Muscle Mass Index; SPPB, Short Physical Performance Battery; Ref, reference category (sarcopenia group).

Multivariate Model 1 adjusted for age, BMI, and alcoholism history. In this model, the protective effects of continuous muscle measures remained significant. Multivariate Model 2 incorporated additional adjustment for sex and CCI score. The results remained robust and largely unchanged. Each kilogram increase in HGS was independently associated with a 1.5% reduction in AC risk (*OR* = 0.985, 95% CI: 0.974–0.996; *p* = 0.006). Similarly, each unit increase in ASM independently predicted a 9.2% risk reduction (*OR* = 0.908, 95% CI: 0.866–0.951; *p* < 0.001). Notably, the presence of sarcopenia emerged as the strongest independent predictor, conferring a 3.56-fold increased odds of AC development (*OR* = 3.564, 95% CI: 2.619–4.860; *p* < 0.001). To directly compare risk across severity categories, we modeled sarcopenia status with sarcopenia as the reference category. Non-sarcopenia was protective (*OR* = 0.264, 95% CI: 0.176–0.394; *p* < 0.001, vs. sarcopenia), whereas severe sarcopenia did not confer additional risk beyond sarcopenia alone (*OR* = 0.883, 95% CI: 0.528–1.472; *p* = 0.633). This threshold effect indicates that meeting the minimal AWGS 2019 diagnostic criteria sufficiently identifies high-risk individuals; further severity stratification does not enhance predictive discrimination.

### Cox regression analysis

To evaluate independent predictors of time to AC development, we constructed multivariate Cox proportional hazards regression models. In univariate analysis, all sarcopenia components demonstrated significant associations with AC risk (Table 4). The co-variables assessed in univariate Cox analysis are presented in Supplementary Table 2. Multivariate Model 1 adjusted for age, BMI, and alcoholism history. In this model, the protective effects of continuous muscle measures remained significant.

**Table 4:** Cox analysis of sarcopenia metrics and time to acute cholecystitis.

Variables	Univariate model		Multivariate model 1		Multivariate model 2	
	**OR [95 %CI]**	***P***	**OR [95 %CI]**	***p***	**OR [95 %CI]**	***p***
HGS (continuous)	0.986 [0.978, 0.995]	0.002	0.989 [0.980, 0.997]	0.011	0.988 [0.979, 0.997]	0.006
ASM (continuous)	0.914 [0.879, 0.950]	< 0.001	0.922 [0.887, 0.959]	< 0.001	0.922 [0.887, 0.959]	< 0.001
SPPB (continuous)	0.958 [0.923, 0.994]	0.024	0.965 [0.929, 1.002]	0.067	0.966 [0.930, 1.003]	0.074
Low HGS (yes)	1.574 [1.268, 1.953]	< 0.001	1.522 [1.226, 1.890]	< 0.001	1.509 [1.208, 1.885]	< 0.001
Low ASM (yes)	1.681 [1.357, 2.082]	< 0.001	1.583 [1.275, 1.966]	< 0.001	1.565 [1.256, 1.951]	< 0.001
Low SPPB (yes)	1.505 [1.208, 1.875]	< 0.001	1.410 [1.129, 1.761]	0.002	1.408 [1.127, 1.759]	0.003
Sarcopenia (yes)	2.626 [2.106, 3.275]	< 0.001	2.400 [1.908, 3.019]	< 0.001	2.419 [1.912, 3.060]	< 0.001
Sarcopenia status						
Non-sarcopenia	0.354 [0.268, 0.469]	< 0.001	0.381 [0.286, 0.508]	< 0.001	0.381 [0.285, 0.510]	< 0.001
Sarcopenia	Ref	Ref	Ref	Ref	Ref	Ref
Severe sarcopenia	0.869 [0.614, 1.230]	0.429	0.843 [0.596, 1.193]	0.334	0.853 [0.603, 1.207]	0.37

Model 1 adjusted for age, BMI, and alcoholism history. Model 2 additionally adjusted for sex and Charlson Comorbidity Index score. HR, hazard ratio; CI, confidence interval; HGS, handgrip strength; ASM, Appendicular Skeletal Muscle Mass Index; SPPB, Short Physical Performance Battery; Ref, reference category (sarcopenia group).

Multivariate Model 2 incorporated additional adjustment for sex and CCI score. The results remained robust and largely unchanged. Each kilogram increase in HGS was independently associated with a 1.2% reduction in AC hazard (*HR* = 0.988, 95% CI: 0.979–0.997; *p* = 0.006). Similarly, each unit increase in ASM independently predicted a 7.8% hazard reduction (*HR* = 0.922, 95% CI: 0.887–0.959; *p* < 0.001). SPPB remained non-significant (*HR* = 0.966, 95% CI: 0.930–1.003; *p* = 0.074). Sarcopenia maintained its position as the strongest predictor (*HR* = 2.419, 95% CI: 1.912–3.060; *p* < 0.001). Consistent with Model 1, with sarcopenia as the reference, non-sarcopenia was protective (*HR* = 0.381, 95% CI: 0.285–0.510; *p* < 0.001), while severe sarcopenia did not confer additional hazard beyond sarcopenia alone (*HR* = 0.853, 95% CI: 0.603–1.207; *p* = 0.370).

## Discussion

Our study represents the first large-scale investigation to demonstrate that sarcopenia, as a multidimensional syndrome defined by the AWGS 2019 criteria, independently predicts the transition from asymptomatic cholelithiasis to acute cholecystitis in older adults. In this retrospective cohort of 1,177 patients aged ≥65 years with incidentally detected gallstones, we found that sarcopenia conferred a 3.56-fold increased odds and 2.42-fold increased hazard of AC development, even after rigorous adjustment for age, BMI, sex, and comorbidity burden. Critically, this predictive capacity was attributable to the syndrome as a whole rather than its individual components alone, and the presence of severe sarcopenia did not incrementally increase risk beyond standard diagnostic thresholds. These findings challenge traditional risk stratification paradigms that rely solely on adiposity-based metrics and suggest that skeletal muscle health may serve as a more physiologically relevant predictor of biliary inflammatory susceptibility in geriatric populations (17, 18).

The observed association between sarcopenia and AC development aligns mechanistically with emerging evidence linking muscle depletion to impaired immunocompetence and heightened inflammatory responses (19–21). Sarcopenia is characterized by chronic low-grade systemic inflammation, evidenced by elevated pro-inflammatory cytokines such as IL-6 and TNF-α, which may prime the gallbladder mucosa for exaggerated inflammatory cascades upon mechanical obstruction (22–24). Furthermore, skeletal muscle serves as a critical reservoir of amino acids and glutamine essential for maintaining gut barrier integrity and supporting acute-phase protein synthesis during physiological stress (25–27). We hypothesize that sarcopenic patients possess diminished metabolic reserve to mount an appropriate anti-inflammatory response when challenged by cystic duct obstruction, thereby lowering the threshold for progression to AC. This notion is supported by our finding that baseline CRP levels were comparable between groups, suggesting that sarcopenia predisposes to *de novo* inflammatory activation rather than merely reflecting pre-existing systemic inflammation.

Our results extend previous literature that has primarily focused on sarcopenia as a postoperative prognostic factor. Prior studies have consistently demonstrated that low skeletal muscle mass predicts increased length of stay, ICU admission, and mortality after AC develops (13, 28, 29). However, by examining the incident risk of AC in a cohort without prior biliary symptoms, our findings position sarcopenia as an upstream determinant of disease initiation rather than merely a downstream marker of frailty. This distinction is clinically significant, as it identifies a window for preemptive intervention before irreversible biliary complications occur. Notably, the nearly three-fold higher prevalence of sarcopenia in the AC group (38.1 vs. 14.0%) suggests that routine sarcopenia screening could reclassify a substantial proportion of asymptomatic gallstone carriers into a high-risk category warranting closer surveillance or earlier cholecystectomy.

The marked discrepancy between the modest discriminative capacity of individual sarcopenia components (AUROCs 0.547–0.584) and the robust predictive power of the composite syndrome (*OR* = 3.564) warrants mechanistic consideration. We attribute this phenomenon primarily to synergistic pathophysiological interactions rather than merely increased prevalence through cumulative abnormality. The AWGS 2019 criteria capture three distinct biological domains—muscle strength reflecting neuromuscular integrity, muscle mass representing metabolic reserve, and physical performance indicating functional integration—that collectively determine physiological resilience. A patient exhibiting isolated low muscle mass may retain sufficient strength and mobility to mount adequate anti-inflammatory responses; conversely, diminished strength alone may be compensated by preserved mass. However, when these deficits coexist, as required by AWGS 2019, the resulting multi-system vulnerability creates a pro-inflammatory, immunocompromised state that substantially lowers the threshold for gallbladder wall inflammation upon stone obstruction. This conceptualization aligns with the threshold effect observed in our severity analysis, wherein severe sarcopenia conferred no additional risk beyond standard criteria, suggesting that meeting the minimal multidimensional threshold sufficiently captures the pathophysiological vulnerability relevant to AC development. Thus, the AWGS 2019 framework succeeds by identifying a high-risk phenotypic state that single-domain assessments fail to detect.

Several clinical implications emerge from our data. First, sarcopenia is a potential modifiable risk factor; resistance training, protein supplementation, and pharmacological interventions have shown promise in reversing muscle loss (30–34). Targeted prehabilitation programs for sarcopenic gallstone carriers could potentially represent a novel primary prevention strategy, potentially reducing emergency admissions and cholecystectomy-related morbidity. Moreover, the identification of sarcopenia as a risk factor may inform decision-making regarding prophylactic cholecystectomy in otherwise asymptomatic patients, particularly those awaiting major surgery or organ transplantation where biliary complications would carry catastrophic consequences.

Nevertheless, important limitations warrant consideration. The retrospective design precludes establishment of temporal causality and may introduce selection bias. Patients undergoing comprehensive sarcopenia assessment likely represent a subgroup with greater healthcare utilization, perceived frailty, or comorbidity burden compared to the broader population of older adults. Our findings may therefore overestimate sarcopenia prevalence and AC risk in unselected populations, and cautious generalization is warranted. Although sarcopenia was assessed at or before the index imaging documenting incident gallstones, and all patients were free of prior biliary symptoms, we cannot exclude the possibility that subclinical, undiagnosed gallbladder inflammation may have influenced muscle metabolism or that shared underlying factors contributed to both conditions. Single-center data from a Chinese cohort limit generalizability, particularly to non-Asian populations where AWGS 2019 cutoffs may require validation. External validation in prospective, multi-center studies across diverse ethnic populations is essential before these findings can inform clinical practice guidelines. While we adjusted for major confounders, residual confounding from unmeasured variables such as dietary patterns, physical activity levels, or microbiome composition cannot be excluded. Additionally, the use of bioelectrical impedance analysis for muscle mass assessment, although aligned with AWGS 2019 recommendations, may be influenced by hydration status and obesity, potentially leading to misclassification. Finally, our cohort was drawn from patients undergoing imaging for various indications, which may overrepresent individuals with greater healthcare utilization and comorbidity burden.

In conclusion, our study establishes sarcopenia as a powerful, independent predictor of acute cholecystitis development in older adults with gallstones, shifting the focus from adiposity to muscle health in biliary risk stratification. These findings suggest a potential paradigm where comprehensive geriatric assessment, including muscle strength and functional performance, may become integral to managing silent cholelithiasis, subject to prospective validation in diverse populations. By identifying sarcopenic patients as a high-risk subgroup, clinicians may implement proactive monitoring and preventive interventions, ultimately reducing the burden of emergency biliary admissions in an aging population.

## Data Availability

The raw data supporting the conclusions of this article will be made available by the authors, without undue reservation.
